# A universal outcome measure for headache treatments, care-delivery systems and economic analysis

**DOI:** 10.1186/s10194-021-01269-9

**Published:** 2021-07-01

**Authors:** Timothy J Steiner, Mattias Linde, Petra Schnell-Inderst

**Affiliations:** 1 Department of Neuromedicine and Movement Science, NTNU Norwegian University of Science and Technology, Edvard Griegs gate, Trondheim, Norway; 2 Division of Brain Sciences, Imperial College London, London, UK; 3 Norwegian Advisory Unit on Headache, Department of Neurology and Clinical Neurophysiology, St Olavs University Hospital, Trondheim, Norway; 4 Tjörn Headache Clinic, Rönnäng, Sweden; 5 Institute of Public Health, Medical Decision Making and Health Technology Assessment, Department of Public Health, Health Services Research and Health Technology Assessment, Medical Informatics and Technology, UMIT – University for Health Sciences, Hall in Tirol, Austria

**Keywords:** Headache disorders, Structured headache services, Cost-effectiveness analysis, Health technology assessment, Outcome measure, Health policy, Global Campaign against Headache

## Abstract

**Background:**

The first manuscript in this series delineated a model of structured headache services, potentially cost-effective but requiring formal cost-effectiveness analysis (CEA). We envisaged a need for a new outcome measure for this purpose, applicable to all forms of treatment, care and care-delivery systems as opposed to comparisons of single-modality treatments.

**Conception and delineation:**

A literature review confirmed the lack of any suitable established measure. We prioritised construct validity, simplicity, comprehensiveness and expression in intuitive units. We noted that pain was the key burdensome symptom of migraine and episodic tension-type headache (TTH), that pain above a certain level was disabling, that it was difficult to put economic value to pain but relatively easy to do this for time, a casualty of headache leading to lost productivity. Alleviation of pain to a non-disabling level would be expected to bring restoration of function. We therefore based the measure on time spent in the ictal state (TIS) of migraine or TTH, either as total TIS or proportion of all time. We expressed impact on health, in units of time, as TIS*DW, where DW was the disability weight for the ictal state supplied by the Global Burden of Disease (GBD) studies. If the time unit was hours, TIS*DW yielded hours lived with (or lost to) disability (HLDs), in analogy with GBD’s years lived with disability (YLDs).

**Utility assessment:**

Acute treatments would reduce TIS by shortening attack duration, preventative treatments by reducing attack frequency; health-care systems such as structured headache services would have these effects by delivering these treatments. These benefits were all measurable as HLDs-averted. Population-level estimates would be derived by factoring in prevalence, but also taking treatment coverage and adherence into account. For health-care systems, additional gains from provider-training (promoting adherence to guidelines and, therefore, enhancing coverage) and consumer-education (improving adherence to care plans), increasing numbers within populations gaining the benefits of treatments, would be measurable by the same metric.

**Conclusions:**

The new outcome measure expressed in intuitive units of time is applicable to treatments of all modalities and to system-level interventions for multiple headache types, with utility for CEA and for informing health policy.

## Introduction

The first manuscript in this themed series noted the high ill-health, disability and economic burdens arising from headache disorders worldwide, which persist despite the existence of effective treatments [1]. It put the blame on widespread failures of health services to deliver these treatments to those who might benefit, failures attributable to lack of awareness, lack of political will and lack of an evidence-based solution [1]. The authors addressed all three of these as barriers to be surmounted, ultimately proposing a model of structured headache services as the health-care solution [1]. They predicted, with some evidential support [2, 3], that this model would be cost-effective and might, potentially, be cost saving if appropriately implemented, but they recognised that these predictions required testing in formal cost-effectiveness analysis (CEA) [1].

Here lay a difficulty: on what outcome measure(s) might economic evaluation of headache services, as opposed to single-modality treatments such as acute or preventative drugs, be based? Previous economic evaluations have used both disease-specific clinical outcomes and generic measures [4–11], but choice among the former has been restricted almost exclusively to those used and reported in randomised clinical trials (RCTs), which are rarely designed to support economic evaluation.

Our purposes in this manuscript were first to review the literature for candidate measures in common use that might serve the requirements of economic evaluation; second, subject to findings, to develop a new measure applicable equally to all forms of treatment, care and care-delivery systems for headache regardless of type; and third to demonstrate its broad utility in these applications.

## Review of potential candidate measures

### Literature search

We did not review the literature systematically for all outcome measures applied to headache care: rather, we judged that our purpose would be better served by a more focused review. We limited it to efficacy measures previously employed in assessing treatments of migraine and/or tension-type headache (TTH), these being the principal headache disorders for which headache services cater [1]. Our envisaged setting was a world in which large numbers of people are affected by headache but have limited treatment options because they live either in developing countries where health-care resources are generally limited or in developed countries where health care for headache has low priority [1, 2, 12]. Therefore, we further limited the review to treatment that would not require consultation with a physician. With this in mind, we looked specifically at RCTs of acetylsalicylic acid (ASA), a choice driven by several considerations. ASA is a first-line treatment with proven efficacy for both migraine and TTH [13–17], it is listed by the World Health Organization (WHO) as an essential medicine for treatment of acute migraine [18], and it is readily available at low cost worldwide.

Also in mind was that only measures expressible in intuitive units, to which economic value could be assigned, would be suitable for purpose. This ruled out a range of multifactorial patient-reported outcome measures (PROMs) [19–25] including the Headache Impact Test (HIT-6) [20] and Headache Under-Response to Treatment (HURT) questionnaire [25], generic [26–28] and disease-specific quality-of-life measures [29, 30] and measures based on WHO’s Classification of Functioning, Disability and Health (ICF) [31–33]. It did not rule out measures based on the Disability-Adjusted Life Year (DALY) since this was developed expressly for CEA and to inform health policy and priority-based decision making [3].

Finally, we limited the review to a 20-year period from 1988, when the International Classification of Headache Disorders (ICHD) first introduced operational and widely accepted diagnostic criteria for migraine and TTH [34]. This period saw the development and introduction of triptans, and drug-company sponsorship not only of RCTs in which ASA was a comparator but also of RCTs comparing ASA with placebo. By the end of this period, this activity had effectively ceased.

The search included MEDLINE and EMBASE along with the Cochrane Library. We supplemented it by reference to recommendations in the two sets of clinical trials guideline published by the International Headache Society (IHS), in migraine [35] and TTH [36].

It found ten studies describing efficacy measures, seven in migraine [37–43] and three in TTH [44–46]. All recruited from outpatient settings and most (all but one of the TTH studies [45]) included patients with headache at base line of at least moderate intensity.

### Commentary

Headache relief after 2 h (HR2) was the preferred endpoint in migraine trials. It was used as the primary outcome measure in five [37, 39, 41–43] and secondary in one [38], and defined in all in accordance with IHS clinical trials guidelines [35] as reduction in headache pain from moderate or severe at base line to mild or no pain at 2 h. Sustained headache relief over 24 h (SHR24) was defined, also in accordance with IHS guidelines [35], as HR2 without, then, use of rescue medication or headache recurrence within 24 h. Five trials reported SHR24 or allowed its calculation by reporting HR2, recurrence rate and use of rescue medication [38, 39, 41–43]. Six trials reported pain-freedom (no pain) at 2 h (PF2) as a secondary endpoint, either directly or by inference from visual analogue scale (VAS) measurements [38–43]. Less frequently used were sustained pain-freedom over 24 h (SPF24), pain intensity difference (PID) calculated as pain intensity on a VAS at base line minus pain intensity at various later times, the sum of PIDs (SPID) recorded at multiple time points and weighted according to the time between observations, and functional impairment (disability) assessed subjectively.

All three TTH studies [44–46] used pain relief, as a change from base line, as the primary endpoint, but defined it differently: at different times and by different means, either on a verbal rating scale (VRS) or on a VAS, sometimes expressed relatively rather than in absolute terms. All studies assessed PID at intervals after treatment, but over different total time periods. Less frequently reported were SPID (although one TTH study used SPID over 4 h as the primary endpoint [44]), use of rescue medication and functional impairment over 24 h.

Of these measures, HR2 has obvious utility. In the migraine trials it was achieved by about half of ASA users, in line with the general perception that a similar proportion with migraine in the general population could effectively self-manage [1] (although people recruited from outpatient settings might not be representative of these). HR2 also distinguished clearly between active and placebo treatments in the trials. However, its 2-hour timeframe in the context of episodes lasting 4–72 h untreated [34] is too short to express efficacy fully. In TTH, pain relief at 2 h (PR2) may be analogous to HR2, but with a limitation (discussed later): it cannot be universally applied, being observable only in those with at least moderate pain at base line while pain in TTH may, often, be only mild [34]. PR2 was achieved in the two relevant TTH trials by about three quarters of ASA users [44, 46], with, again, clear distinction between active and placebo treatments.

Pain-freedom may be the outcome most desired by people treating either disorder, and the IHS guidelines for clinical trials in migraine recommend PF2 as primary efficacy endpoint [35]. But PF2 in the migraine trials we reviewed was uninformative about treatment effect in more than three quarters of ASA users because they did not achieve it, although many of these might nonetheless have had worthwhile benefit [47]. Again, it covers too short a timeframe to express efficacy fully. The same endpoint, reported as the conceptually approximate “total” PR2 in the TTH trials we reviewed, is recommended as primary efficacy endpoint by the IHS guidelines for clinical trials in TTH [36], but remains similarly limited.

PID equates reductions in pain intensity (whether absolute or proportional) regardless of its initial level. Its inbuilt assumptions about the pain intensity continuum, and that perceived change is independent of base line, render it conceptually dubious. Thus, for example, on a VAS or NRS, PID attaches similar meaning to absolute reductions from 10 to 7 (remaining severe), from 5 to 2 (moderate becoming mild) and from 3 to 0 (pain resolution). Expressed proportionately (often as reduction by 50 %), it gives equal weight to reductions from 8 to 4 and from 4 to 2. Further, when PID measurements are made serially, each is not independent of the one before. PID therefore has validity only at a single pre-specified time point. SPID, an integrating measure over time, circumvents the last problem but is highly cumbersome, requiring multiple timed measurements if applied over a timeframe long enough to express efficacy adequately.

While use of rescue medication is a measure reflecting primary inefficacy, and expresses an important dimension of outcome, it is applicable only to subsets experiencing this: in the trials, about 60 % taking ASA for migraine and 85 % for TTH did not require rescue. Conversely, while headache recurrence is also an important outcome for those experiencing it, recurrence within 24 h as an endpoint is possible only in those who initially respond. SHR24, in either migraine or TTH (and defined in the same way in both), takes account of both recurrence and use of rescue medication while also capturing primary efficacy. It is measurable in everybody with at least moderate base-line pain (including all randomised patients in most RCTs – an important consideration) by observations at two time points only. While it represents an imperfect outcome [47], several economic studies have used it as the option that best reflected treatment success [4, 7, 8].

None of these disease-specific outcome measures embrace *disability*. Both sets of IHS guidelines recommend assessing disability by VRS as secondary outcome measures [35, 36]. For most people, the immediate objective of treating acute episodes of migraine or TTH, the two causes of most of the headache-attributed ill-health burden [48–52], is to relieve symptoms, of which headache is usually predominant [15]. Preventative drugs have the same but more distanced objective, relieving symptom burden by reducing future episode frequency [15]. In either case, the purpose beyond alleviating pain is to minimise disability, a key consequence of pain [53]: acute treatment is taken in the hope of restoring normal function as quickly as possible, preventative treatment to avert its loss. This suggests that disability should be the focus of outcome measurement [53, 54], particularly since disability is the presumed cause of lost productivity, the major contributor to economic cost [55].

It should be noted that this presumption is not a given [56]. At issue are the meaning of *disability*, to which we make reference later, and the relationship between disability (however defined) and lost productivity, which is complex [57]. Nevertheless, a disability-based outcome measure could serve all purposes: comparative evaluation of headache treatments of all types, effectiveness assessment of care-delivery systems, and economic analysis. Of the trials, only a minority reported functional impairment [40, 42, 43, 46], always as a subjective assessment: in migraine, on either a 4-point (“none”, “mild”, “moderate” or “severe”) [43] or a 5-point VRS (“able to perform all activities”, “daily activities require a little additional effort”, “daily activities require some additional effort”, “daily activities require a great deal of additional effort” or “unable to perform daily activities”) [42]. These, applied retrospectively to the attack as a whole, were unsophisticated assessments; lacking any time dimension, they could not be useful in comparisons of treatments of different modalities or in the context of economic analysis.

### Conclusions

In short, no outcome measure in common use could meet all requirements. More specifically, to our earlier question (“On what outcome measure(s) might economic evaluation of headache services, as opposed to single-modality treatments such as acute or preventative drugs, be based?”), asked in an envisaged but real world in which many people are affected by headache but have limited treatment options [1], none of these is the answer.

## Development of a new measure

### Conception and delineation

We prioritised four properties of a new measure: construct validity, of course, but also simplicity, comprehensiveness and expression in intuitive units. We also took the view that a universal outcome measure must in some manner take account of clinically important adverse events (AEs) reasonably attributable to the intervention.

As a starting point, with regard to construct validity, we noted that pain was the key burdensome symptom of both migraine and episodic TTH, and that pain above a certain level was disabling [52, 53]. We recognised also that endpoints expressing key symptoms were not always amenable to economic analysis: it was difficult, for example, to put an economic value to pain. On the other hand, it would be *relatively* easy to do this for *time*, very often a casualty of headache leading to the reportedly high indirect costs of lost or reduced productivity [55, 58, 59]. Alleviation of pain to a non-disabling level would be expected to bring restoration of function; the sooner this was achieved, the greater the recovery of useful (productive) time that would otherwise be lost.

With regard to comprehensiveness, we also noted that, to make comparisons across diseases, generic measures of health outcomes were needed. The DALY, used by WHO and the Global Burden of Disease (GBD) study [1, 48–51]), is a generic summary measure of *lost health*, having utility in relative assessments of health benefits gained by interventions. DALYs are the sum of years of life lost to premature mortality (YLLs) and years lost to disability (YLDs); because headache does not reduce life expectancy, DALYs here equate to YLDs. GBD2013 derived disability weights (DWs: valuations on a 0–1 scale, where 1 = full health) for health states consequent upon diseases (including the ictal states of migraine and TTH) [60] from a worldwide survey eliciting comparative judgements about these health states. YLDs attributable to health states are calculated as the product of time in the health state and its DW. While this became the basis for estimating global burdens attributable to each disease, and ranking diseases accordingly [48–51], it also, along with SHR24, provided us with the foundations on which to develop a universal outcome measure for headache.

With reference to earlier comments, we say more later about the meaning of *disability* in the specific context of YLDs and DWs (see Discussion). In the following, we use the term with this meaning.

### Hours lost to (or lived with) disability (HLDs)

We based the measure on time spent in the ictal state (TIS) of either migraine or TTH, expressed as total time (tTIS) or proportion of total time (pTIS). In the context of RCTs looking at single acute episodes, TIS was expressed simply by attack duration with or without treatment. In the more general contexts of individual and population health, tTIS and pTIS were expressed for a defined period of time in the former case as the product of mean duration and mean attack frequency and in the latter case as D*F*prevalence, D and F here representing the population means of attack duration and frequency. For each disorder (migraine or TTH), the absolute impact on health was expressed, in units of time, as tTIS*DW during a prescribed period and the relative impact, as a proportion, as pTIS*DW. If the time unit was hours, the most logical and practical, tTIS*DW yielded hours lost to disability (HLDs), in analogy with YLDs.

### HLDs averted

Acute treatments would diminish (avert) HLDs by shortening attack duration, preventative treatments by reducing attack frequency; health-care systems such as structured headache services [1] would have these effects or would supplement them by delivering or enhancing delivery of these treatments. These benefits would all be measurable as HLDs averted.

### Utility assessment

To demonstrate the measure’s broad utility, we theoretically modelled its application in acute and preventative therapies, and in the evaluation of a service-delivery model such as structured headache services [1] providing migraine care. In each case, we modelled the effects of the intervention on either attack duration or attack frequency, at individual or population levels.

**Fig. 1:**
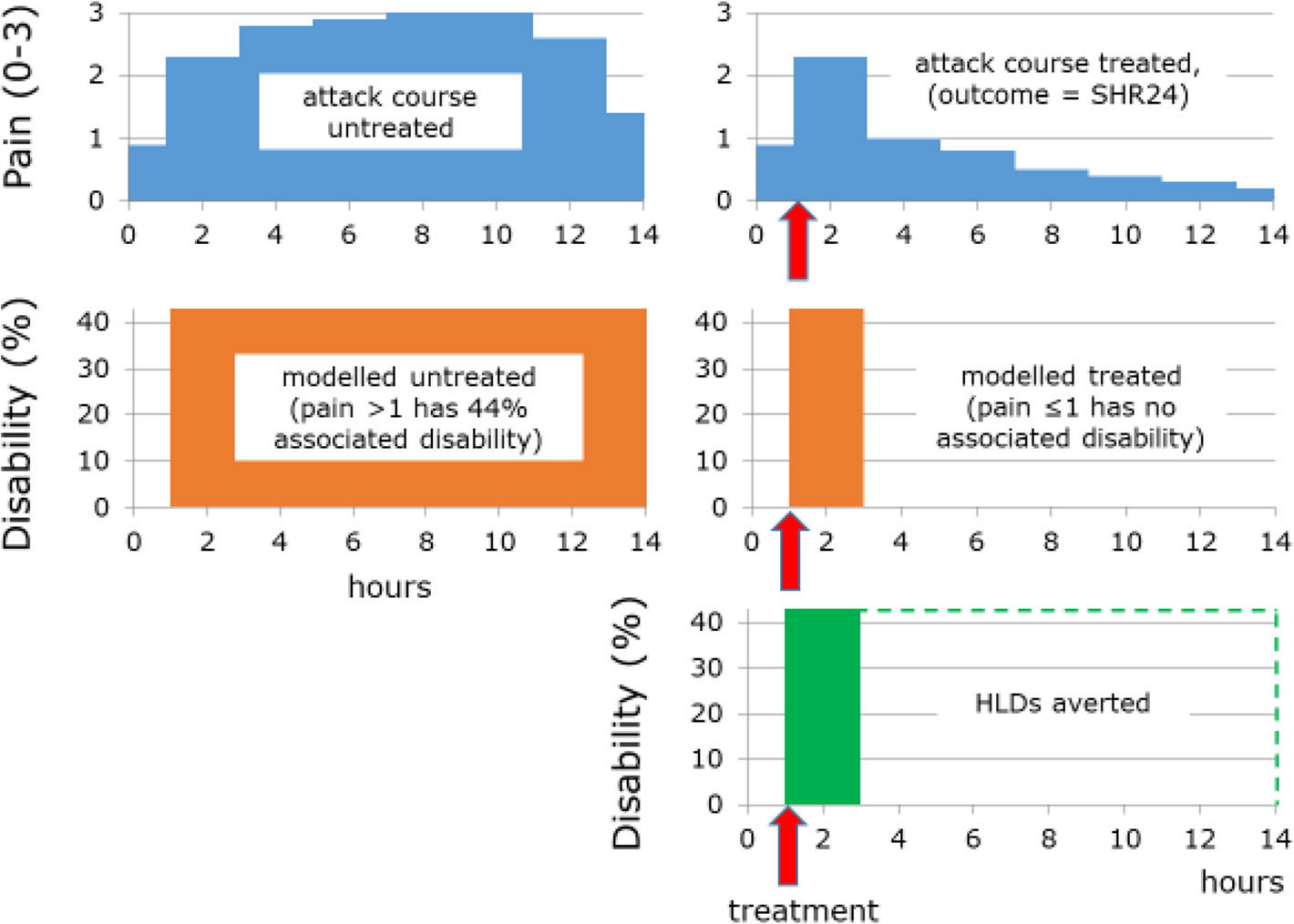
Figurative depiction of how the outcome measure applies to acute therapy over the course of a single migraine attack in an individual (see text for explanation)

Figure 1 is a figurative depiction of how the outcome measure applies to *acute therapy* for migraine in an *individual*. On the left is the imagined course of an untreated attack of 14 hours’ duration. From onset, pain would increase to its maximum over several hours, plateau, then decline rapidly as the attack ended spontaneously. For the period of 13 h during which pain was greater than mild (> 1 on the scale 0–3), disability (depicted below on the left) would be according to the DW for the ictal state from GBD2013 [60] (i.e., 44 %). On the right, acute therapy taken at 1 h and achieving efficacy (i.e., SHR24) would reduce pain to mild within two hours, without recurrence. Disability, again modelled below, would be 44 % until pain was reduced and then zero according to the assumption that mild pain was not disabling [52, 53]. Thus, duration would be reduced from 14 to 3 h, with 11 HLDs averted (bottom right).

*Population* estimates would involve interpreting SHR24 reports from RCTs (for example [17, 61, 62]): acute drugs would reduce pain intensity from disabling levels to non-disabling within 2 h, without recurrence, in the proportion of attacks stipulated by SHR24 as a reported outcome measure. Thus, if treatment was taken at onset, tTIS would effectively be reduced in this proportion of attacks from expected duration to 2 h, where expected duration might be the mean placebo-treated duration in an RCT or the mean derived from population-based data. An imaginary numerical example for a population is given in Table 1.

**Table 1:** Imaginary numerical example of acute treatment effect in a population

Mean untreated attack duration (uD)	12 h
Successfully-treated attack duration (tD)	2 h
Change in mean time in ictal state (dTIS)	uD-tD = 12 − 2 = 10 h
Number of attacks treated (nA)	100
Probability of treatment success (SHR24) (pE)	50 %
Change in total time in ictal state (dtTIS)	dTIS*nA*pE = 10*100*50 % = 500 h

For *preventative treatments*, tTIS would be reduced directly in proportion to effect on attack frequency. Table 2 is an imaginary numerical example for an individual, for whom untreated (uF) and treated (tF) attack frequencies were observable; at population level it would be necessary to factor in estimates of treatment effect (dF) and its probability (pE) derived from RCTs.

**Table 2:** Imaginary numerical example of preventative treatment effect in an individual

Untreated attack frequency (uF)	60/year
Treated attack frequency (tF)	30/year
Change in attack frequency (dF)	30/year
Attack duration (D)	14 h
Change in total time in ictal state (dtTIS)	dF*D = 30*14 = 420 h/year

All of these effects would ultimately be expressed in HLDs averted by introduction of the factor DW [60]. For *acute treatments*, in the context of a single attack, this is illustrated in Fig. 1. For multiple attacks over time, HLDs averted would be dtTIS*DW, with the assumption that treatment was taken before or as soon as headache became disabling so that there was no measurable health loss before treatment. The proportion pE deemed to be successfully treated could be reducible to take account of clinically important AEs according to their frequencies by negating success when they occurred. (This adjustment might be over-conservative, but is an option available according to purpose. It would not satisfactorily account for serious AEs, which always require separate recording.) For *preventative treatments*, effects would be expressed in HLDs averted through the same product dtTIS*DW, again reducible, should the purpose require it, by the proportion of treatment discontinuations due to AEs.

Should both acute and preventative drugs be taken, interaction would be duly taken account of by projecting the potential effect of the former alone onto attacks not averted by the latter.

*Population-level* estimates would be derived by factoring in prevalence, but also taking treatment coverage and adherence into account as well as efficacy. For *health-care-system interventions*, effects should be expressed through gains in these (more people receiving and taking appropriate treatments) as the consequences of provider-training (enhancing care coverage) and consumer-education (improving adherence) [1], each by specifiable amounts. Improvements in either would proportionately increase numbers within populations gaining the benefits of treatments, and therefore the total population-level benefits measurable by the same metric.

For TTH, the measure is similarly applied, but with the limitation arising from the fact that TTH may only be mild [34] – more commonly so than migraine. Because the DW for the ictal state of TTH is much lower (0.037 as opposed to 0.441 for migraine [60]), HLDs are far fewer. These limitations are discussed below.

## Discussion

This presentation has described the conception and delineation of a new universal outcome measure applicable to treatments of all modalities of headache of multiple types (migraine or TTH, but also of other types manifesting as attacks definable in terms of duration and frequency) and expressed in intuitive units of time. Its development relied on *disability* as estimated in the GBD studies [48–51]. The measure was applied in theoretical scenarios to both acute and preventative migraine treatments, to individuals and populations, and to systems designed to deliver these treatments, with all outcomes expressed by the same single metric rooted in the YLD.

What was the need? Multiple outcome measures existed already for treatments of migraine and TTH, and several were widely accepted, though not, perhaps, with universal agreement [47, 63]. These performed the important clinical function of allowing comparisons of treatments to inform choice. Where they fell short was in comparisons of treatments of different modalities. In the context of economic analysis, with the purpose of valuing interventions of different types relative to each other, existing measures were applied with difficulty. When interventions were at the level of service organisation and delivery rather than provision of single-modality treatments (such as drugs), they were unhelpful. When the purpose was to inform prioritisation within health policy of resource allocation across diseases, they failed altogether. The need we recognised was defined by this last purpose, but all purposes are served by the new measure.

HLDs as the unit of our measure are calculated by introducing DW for the ictal state (of either disorder) as a factor. In the context of interventions only for migraine, our modelling example, DW is a constant (set at 0.441 by GBD2013 [60]). It is not, therefore, essential for comparisons between interventions at whatever level (acute, preventative or care-delivery system). However, when other headache types are also of interest, with different DWs, and certainly when other (non-headache) diseases are comparators of interest, DWs are the essential enabling factor, as they are for YLDs [48–51].

However, despite their name, DWs reflect public preferences for health states (and therefore ill health in its broadest sense), *not* disability [56, 57, 64]. This does raise a question about the construct validity of our measure, which, we argued, was grounded on disability and its effect on productivity as the key elements of headache-attributed burden against which interventions were or should be directed. Whereas *time* is noted to be a casualty of disabling headache, and, to the extent that time is used productively, this leads to lost productivity, and whereas both absenteeism from work and presenteeism are observed consequences at least of migraine [55, 59], there is a pressing need for empirical evidence showing the relationship between YLDs (and HLDs) and lost productivity. Economic analyses seeking to incorporate indirect costs, and especially those doing so to assess the value of headache treatment, require knowledge of the presumed but uncertain relationship between HLDs averted and recovered productivity. Later papers in this themed series go some way to addressing this [56, 57].

HLDs averted are a conservative measure. Several of the assumptions behind its conception and application were conservative. Important among these was that efficacy is all-or-nothing: SHR24 either occurs or it does not. Basing HLDs averted on SHR24 led to conservative effect-estimation in two ways: lesser but possibly still worthwhile effects would not be counted, and no higher value would be attached to greater effects such as sustained pain-freedom. This is justifiable in CEA (conservatism rules in economic analysis), and we would argue that it is not problematic in clinical contexts, where serial assessments of patients are made relatively to assess change. In GBD evaluations, disability is also all-or-nothing: it is at the level stipulated by the DW for the health state (44.1 % for the ictal state of migraine [60]) or it is zero. More contentious is our suggestion that attacks otherwise successfully treated (positive SHR24) but attended by clinically important AEs (but not serious AEs) might optionally be discounted. This may be justified in economic analysis, but clinically appears over-conservative: a more nuanced approach may be more appropriate. Of course, no benefit can be attributed to preventative treatments necessarily discontinued because of AEs.

We should note that among the limitations of this presentation are other assumptions. One, that mild headache is not associated with disability, is a standard assumption [52, 53] not needing justification. A second, that acute treatment is initiated before or as soon as pain becomes disabling, is an assumption necessary to establish a time zero for purposes of effect calculation. It reflects recommended practice [13–15], to be promoted by public education as part of structured headache services [1], and perhaps, therefore, also requires no justification.

Specific limitations, identified earlier, apply to TTH. Because it discounts mild pain, the measure has a more restricted application to TTH than to migraine: TTH is, usually but not always, a mild-to-moderate headache [34]. When base-line pain is mild, the assumption of no disability attributable to it accords zero value to it. In the present context this is of little consequence since mild TTH does not call upon structured headache services [1], or, usually, professional intervention of any sort.

The DW for the ictal state of TTH is much lower than that of migraine: 0.037 as opposed to 0.441 [60]. Of course, this valuation reflects the very fact that TTH is commonly mild. The consequences are that attributable HLDs are far fewer and health care can achieve relatively little in terms of HLDs averted. In other words, the measure is insensitive to TTH. The same is true of estimates of YLDs attributed to TTH in the GBD studies [48–51], but it is a consequence of the public perception of TTH on which its DW is based [60].

## Conclusions

We have described the development of a new universal outcome measure expressed in intuitive units of time and applicable to treatments of all modalities of headache of multiple types. The measure equips economic analysis of interventions, including implementation of structured headache services, for purposes including informing health policy.

## Data Availability

Not applicable.

## Abbreviations

AE: Adverse event
ASA: Acetylsalicylic acid
CEA: Cost-effectiveness analysis
DALY: Disability-adjusted life year
dF: Change in frequency (of attacks)
dTIS: Change in time in ictal state
dtTIS: Change in total time in ictal state
DW: Disability weight
GBD: Global burden of disease (study)
HLD: Hour lost to (or lived with) disability
HR: Headache relief
IHS: International Headache Society
MOH: Medication-overuse headache
nA: Number of attacks treated
PF: Pain-freedom
PID: Pain intensity difference
pE: Probability of treatment success
PROM: Patient-reported outcome measure
RCT: Randomised controlled trial
SHR: Sustained headache relief
SPF: Sustained pain-freedom
SPID: Sum of pain intensity differences
tD: Duration of treated attack
tF: Treated attack frequency
TIS: Time in ictal state
TTH: Tension-type headache
tTIS: Total time in ictal state
uD: Duration of untreated attack
uF: Untreated attack frequency
UK: United Kingdom
VAS: Visual analogue scale
VRS: Verbal rating scale
WHO: World Health Organization
YLD: Year lived with disability
YLL: Year of life lost
